# Role of Fine Needle Aspiration Cytology in Diagnosis of Solitary Thyroid Nodules 

**Published:** 2011

**Authors:** Fazal I Wahid, Sahibzada Fawad Khan, Habib Ur Rehman, Iftikhar Ahmad Khan

**Affiliations:** 1 Department of otorhinolaryngology, Post Graduate Medical Institute, Peshawar, Khyber Pakhtunkhwa Pakistan; 2 Department of otorhinolaryngology, Post Graduate Medical Institute, Peshawar, Khyber Pakhtunkhwa,Pakistan; 3 Department of otorhinolaryngology, Post Graduate Medical Institute, Peshawar, Khyber Pakhtunkhwa,Pakistan; 4 Department of otorhinolaryngology, Post Graduate Medical Institute, Peshawar, Khyber Pakhtunkhwa,Pakistan

**Keywords:** Fine needle aspiration cytology, Histopathology, Solitary thyroid nodule

## Abstract

**Introduction:**

This study was conducted at the Department of ear, nose, throat, head and neck surgery, Post Graduate Medical Institute Lady Reading Hospital Peshawar. The duration of the study was one year from June 17, 2009 to June 16, 2010. The sample size was 82 patients with solitary thyroid nodule, fulfilling the inclusion criteria. After taking detailed history, thorough examination, relevant investigation and informed consent fine needle aspiration cytology was performed in all cases by the same cytopathologist. Thyroid surgery was performed and specimens were examined by the same histopathologist. The statistical analysis was performed using the statistical program for social sciences (SPSS version 11).

**Materials and Methods:**

Our study included 82 cases consisting on 57 female and 25 male, with female: male ratio of 2.28: 1.The age of the patients was ranged from 16-65 years with mean age of 42.56 + S.D 11.60 years. Most of the patients presented in 3^rd^ and 4^th^ decade followed by the 5^th^ and 2^nd^ decade. The diagnostic yield of Fine Needle Aspiration Cytology (FNAC) in this study was accuracy 82.92%, sensitivity 88.09%, specificity 77.50% and positive predictive value was 80.43%.

**Results:**

One hundred twenty six patients entered the study among which 77 (61%) were female and 49 (39%) male. Mean age was obtained as 26.9 ± 7.7 yrs. Up to 79.4% of patients had complaints concerning the cosmetic outcomes, 39.7% with respiratory and 4.8% with olfactory problems. The reason to sue the physician had a significant relationship with the patients’ age and sex, and also with the surgeons’ experience.

**Conclusion:**

FNAC has key rule in diagnosis of solitary thyroid nodule because it is safe, minimally invasive and cost effective diagnostic tool.

## Introduction

Frequency of thyroid disease is common in Pakistan and solitary thyroid nodule presents a significant diagnostic dilemma for the treating surgeon (1). Thyroid nodule occurs in 4-7% of the population (2). Malignant tumors of thyroid gland represent less than 0.5% of all cancers (3). Although solitary thyroid nodules are common in females, they are more likely to be malignant in males (4). Different imaging techniques are now used for pre operative diagnosis of solitary thyroid nodule like radio nucleotide scanning, high resolution ultrasonography etc. but fine needle aspiration cytology is regarded as the single and most cost-effective procedure (5). Fine needle aspiration cytology of malignant thyroid nodules reported to have sensitivity and specificity ranges from 65-98% and 72-100% respectively (6). Although there is a large body of world literature claiming the accuracy and usefulness of thyroid cytology, there is also evidence of showing possible limitations and pitfalls of this procedure (7). Fine needle aspiration cytology of thyroid gland is now a well established, first line diagnostic test for the evaluation of diffuse thyroid lesion as well as of solitary thyroid nodule with main purpose of confirming benign lesion and by reducing unnecessary surgery (8). Virtually any disease of thyroid can be presented as a nodule and it is not usually possible to distinguish between benign and malignant thyroid nodule by any non invasive procedure (9). Use of FNAC for Thyroid enjoys unmatched popularity as it is predominantly related to the cosmetic complication and technical difficulties of thyroid surgery and relatively small number of true neoplasms in patients with thyroid nodules (10). The main goal of evaluating these nodules by FNAC is to identify nodules with malignant potential and get prompt management of them, considering the limitations of open biopsy and advantages of FNAC.

## Materials and Methods

This descriptive study was conducted at the department of Ear, Nose, Throat, Head and Neck Surgery, Postgraduate Medical Institute Lady Reading Hospital Peshawar from June 17, 2009 to June 16, 2010. It included 82 cases of solid solitary thyroid nodule fulfilling inclusion criteria. *Inclusion criteria: *1. Both male and female patients.2. All age groups.3. Solitary thyroid nodule. 

*Exclusion criteria: *1. Non-thyroidal neck masses. 2.Diffuse goiter. 3.Multinodular goiter. This study was approved by hospital Ethical committee. The diagnostic criterion for solitary thyroid nodule was the triple assessment including clinical, radiological and tissue diagnoses. A well informed consent was taken. The technique, risks, benefits, results and associated complications of the procedure were discussed with all patients. A detailed history was taken and the patient was thoroughly examined. Mucosal lining of upper aero-digestive tract was examined and systemic examination was also carried out. Routine investigations were performed in all cases. Ultrasonography, radioiodine scan, thyroid function tests, computed tomography, MRI and endoscopy were done when indicated. Fine needle aspiration cytology was performed in all cases by the same cytopathologist. Thyroid surgery was performed and specimens were examined by the same histopathologist. The statistical analysis was performed using the statistical program for social sciences (SPSS version 11). The frequencies and percentages were presented for qualitative variables and Mean + SD were presented for quantitative variables. All the relevant information was documented on a pre-designed proforma. Sensitivity, specificity, positive predictive value and negative predictive value were calculated for fine needle aspiration cytology taking histopathologic examination as gold standard.

## Results

Our study included 82 cases of solitary thyroid nodule fulfilling inclusion and diagnostic criteria. There were 57 females and 25 males, with female: male ratio of 2.28: 1. The age of the patients was ranged from 16-65 years with mean age of 42.56+S.D 11.60 years. Most of the patients presented in 3^rd^ and 4^th ^decade followed by the 5^th^ and 2^nd^decade (Graph 1). The main complaints of these patients were neck swelling (100%), vocal cord palsy (6.09%), breathing difficulty (4.87%) and dysphagia (Table 1). The size of the thyroid nodule ranged from 2 - 7.2 cm with mean 4.40 +/- S.D 1.93 cm. The solitary nodule was found mainly in right lobe of thyroid (64.63%) and the least involvement was of thyroid isthmus. 

**Fig 1:**
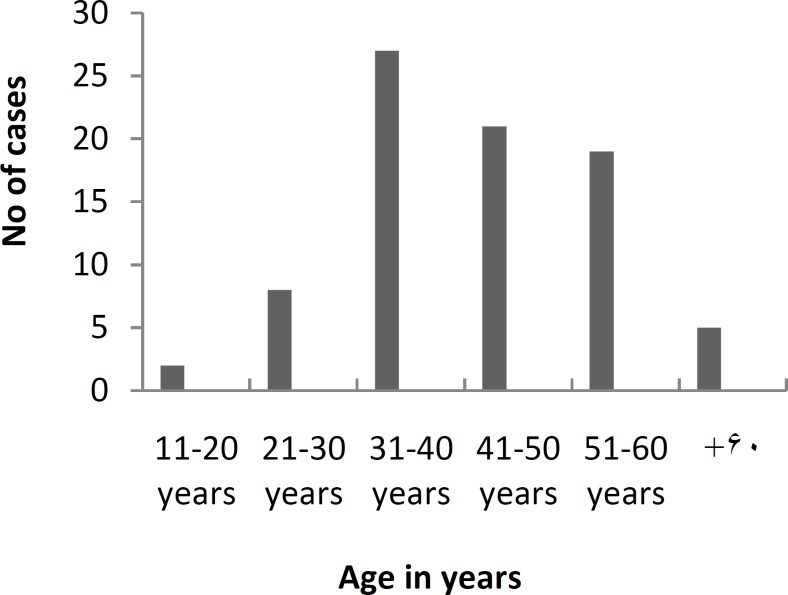
Age-wise distribution of patient (n=82).

**Table 1:** Clinical features of patients (n=82).

**S. No**	**Symptom/ Sign**	**NO. of cases**	**Percentage**
1	Neck Swelling	82	100%
2	Vocal Cord Palsy	05	6.09%
3	Difficult Breathing	04	4.87%
4	Dysphasia	03	3.65%
5	Hoarseness	02	2.43%
6	Weight Loss	02	2.43%

In this study FNAC of solitary thyroid nodule revealed that 42 cases (51.21%) were nodular goitre, 13 cases (15.85%) benign cyst among benign lesions while 13 cases (15.85%) were follicular carcinoma, 8 cases (9.75) papillary carcinoma and 2 cases were suspicious of neoplasm(Table 2). In our study histopathological findings of thyroid nodule were as 40 cases (48.78%) of colloid nodule, 10 cases (12.19%) benign thyroid cyst and one case of Hashimoto’s thyroiditis among benign conditions while neoplastic lesions were 13 cases (15.85%) follicular adenoma, 10 cases (12.19%) colloid adenoma followed by papillary carcinoma 3 cases (3.65%) and 2 cases of follicular carcinoma (Table 3).

**Table 2:** FNAC of thyroid nodule (n=82).

Diagnosis on FNAC		Patients		
		No. of cases		% Age
**Non neoplastic lesions**	Nodular goitre Benign cyst Lymphocytic thyroiditis	42 13 3	58	70.73
				
**Neoplastic lesions**	Follicular carcinoma Papillary carcinoma Hurthle cell lesion Suspicious of neoplasm	13 8 1 2	24	29.26
Total patients			82	100%

**Table 3:** Histopathology of thyroid nodule (n=82).

**Diagnosis on histopathology**		Patients		
		No. of cases		% Age
**Non neoplastic lesions**	Solitary colloid nodule Benign thyroid cyst Ch. Lymphocytic thyroiditis Hashimoto's thyroiditis	40 10 2 1	53	64.63
				
**Neoplastic lesions**	Follicular adenoma Colloid adenoma Hurthle cell adenoma Follicular carcinoma Papillary carcinoma	13 10 1 2 3	29	35.36
**Total patients**			82	100%

The diagnostic value of FNAC in this study was as follows: 37 cases (45.12%) were true positive, while 31 cases (37.80%) were true negative. In this study false positive cases were 9 (10.97%), 5 cases were follicular neoplasm on FNAC while on histopathology they turned out to be benign thyroid diseases, 3 cases were papiillary carcinoma which had been diagnosed benign thyroid cyst on biopsy (Table 4). 

In our study 5 cases (6.09%) were false negative, 3 cases were benign thyroid diseases which were diagnosed papillary carcinoma on histopathology, 2 cases (2.43%) were diagnosed aslymphoma and follicular adenoma on histopathology (Table 5).

**Table 4:** Table of frequency of diseases in this study (n=82).

**Gold standard test (BIOPSY)**			
**Test result (FNAC)**	**Disease**	**No disease**	**Total**
Positive	**37**	**9**	**46**
Negative	**5**	**31**	**36**
Total	**42**	**40**	**N=82**

**Table 5:** Diagnostic comparison between FNAC and histopathology for solitary thyroid nodule (n=82).

**Cytological diagnosis**	**Histological diagnosis**	**Frequency**		**Remarks**
Follicular neoplasm Hurthle cell lesion Papillary carcinoma Suspicious neoplasm	Follicular adenoma Follicular carcinoma Hurthle cell adenoma Papillary carcinoma Colloid adenoma	15 11 1 7 3	37	True Positive
				
Follicular neoplasm Papillary carcinoma Suspicious of neoplasm	Nodular goitre with hyperplasia Ch. Lymphocytic thyroiditis Benign thyroid cyst Hashimoto's thyroiditis	3 2 3 1	9	False Positive
				
Nodular goiter Benign cystic lesion of thyroid Ch. Lymphocytic thyroiditis Hushimoto’s thyroiditis	Solitary colloid nodule Benign thyroid cyst Ch. Lymphocytic thyroiditis Hashimoto's thyroiditis	19 9 2 1	31	True Negative
				
Nodular goiter Benign cystic lesion of thyroid Ch. Lymphocytic thyroiditis Nodular goitre with hyperplasia	Papillary carcinoma Papillary carcinoma Lymphoma Follicular adenoma	2 1 1 1	5	False Negative
Total Patients			82	

**Table 6:** Diagnostic yield of FNAC in diagnosis of solitary thyroid nodule (n=82).

**Accuracy**	**Sensitivity**	**Specificity**	**PPV**	**NPV**
82.92%	88.09%	77.50%	80.43%	86.11%
				

## Discussion

FNAC-based detection of solitary thyroid lesions remains challenging, in spite of tireless efforts to establish cytologic and clinical criteria for diagnosing follicular neoplasms and distinguishing between benign and malignant lesions (11). Nonetheless, it is widely accepted that presently, FNAC is the best and most reliable diagnostic tool for use in the preoperative management of patients with such lesions. Thyroid nodule is more common in females than males. In this study there were 57 females and 25 males, with female: male ratio of 2.28: 1, which is comparable to the studies conducted nationally and internationally (12). In this study most of the patients presented in 3^rd^ and 4^th ^decade which is in accordance to the study of Bukhari and collegues (13). In this study the FNAC finding was as follows: 58 cases (70.73%) had non neoplastic lesions which in accordance to study of Korah (14) reporting benign lesions 69%, while in some of the studies benign lesions were found in 50% cases (15). Nodular goitre was the most common finding among the benign lesions (51.21%) which agrees with studies of Gupta (16) revealed 39 cases (52%) as colloid nodular goitre and Saddique (17). reported thirty cases (50%) as nodular goiter. The next common FNAC finding among benign lesions was benign cyst in 13 cases (15.85%) which is at variance from study of Abu-Salem having thyroid cysts in 43 cases (8.3%) (18). The malignant diseases in this study were 29.26% which is comparable to the study of Gupta (16) having malignant lesion 26% and Baloch study having malignant lesions 29% (n-110) (19). Among the malignant diseases follicular carcinoma was on top accounting 15.85% which is different from study of Pai where malignancy was found in 15 cases (23%) (20). On histopathology non neoplastic lesions were 64.63% and neoplastic lesions were 35.36% while in Mehmood (21) study histopathology revealed non neoplastic lesions 79.49% and neoplastic lesions 20.51%. Among the neoplastic lesions on histopathology follicular adenoma was found in 13 patients (15.85%) while in Tabaqchali (22) study follicular adenoma was found in 60 patients (25.10%). On FNAC 8 cases (9.75%) were diagnosed as malignant and on histopathology they were confirmed benign nodular goitre and one case was suspicious on FNAC and was confirmed as Hashimoto’s thyroiditis on histopathology which is comparable to the study of Gharib (23) who reported a false-negative rate of 1% to 11%, a false-positive rate of 1% to 8%.In this study the diagnostic yield of FNAC for solitary thyroid nodule including sensitivity, specificity, PPV and NPV were 88.09%, 77.50%, 80.43%and 86.11% respectively. In the literature the diagnostic yield of FNAC has different values ranging from 50% to 95%. Kumar revealed sensitivity and specificity of 77% and 100% respectively (24). In Moosa study the yield of FNAC was as follows: sensitivity 77.7%, specificity 98.9%, positive predictive value 87.5% and negative predictive value 97.8%. (25) Similarly Abu-Salem studied specificity of 99% and a sensitivity of 93% (18). Tariq reported sensitivity 75%, specificity 97.6%, PPV 85.71% and NPV 95.34% (26). Saddique showed in his study sensitivity of 75%, specificity of 95.83%, positive predictive value of 81.81% and negative predictive value of 93.81% (17). Likewise Alam reported sensitivity of 100% and specificity of 95.12% (27) My results are lesser than the study of Korah (14) who reported 88%, 98%, 100% and 100%, for sensitivity, negative predictive value (NPV), specificity and positive predictive value (PPV) respectively. The outcome of FNAC in Mehmood study showed sensitivity 79.17% and specificity 91.40% (21). In my study the accuracy of FNAC was 82.92% which is comparable to the studies of Bukhari (13) having accuracy 87%, Pai accuracy 89% (20). However accuracy of my study is greater than Gupta study revealed accuracy of 13.3% (16).

## Conclusion

FNAC has key rule in diagnosis of solitary thyroid nodule because it is safe, minimally invasive and cost effective diagnostic tool for preoperative assessment of patients with thyroid nodule to help the surgeon in management of these nodules. 
